# Feeding on a *Bartonella henselae* Infected Host Triggers Temporary Changes in the *Ctenocephalides felis* Microbiome

**DOI:** 10.3390/pathogens12030366

**Published:** 2023-02-22

**Authors:** Charlotte Moore, Erin Lashnits, Pradeep Neupane, Brian H. Herrin, Michael Lappin, Marcos Rogério André, Edward B. Breitschwerdt

**Affiliations:** 1 Intracellular Pathogens Research Laboratory, Department of Clinical Sciences, College of Veterinary Medicine, North Carolina State University, Raleigh, NC 27607, USA; 2 School of Veterinary Medicine, University of Wisconsin-Madison, Madison, WI 53706, USA; 3 Department of Microbiology and Immunology, The University of North Carolina at Chapel Hill, Chapel Hill, NC 27599, USA; 4 Department of Diagnostic Medicine/Pathobiology, Kansas State University, Manhattan, KS 66506, USA; 5 Center for Companion Animal Studies, Department of Clinical Sciences, Colorado State University, Fort Collins, CO 80523, USA; 6 Vector-Borne Bioagents Laboratory (VBBL), Department of Pathology, Reproduction and One Health, Faculdade de Ciências Agrárias e Veterinárias, Universidade Estadual Paulista (FCAV/UNESP), Jaboticabal 14884-900, Brazil

**Keywords:** *Bartonella henselae*, *Ctenocephalides felis*, flea, microbiome, wolbachia, vector microbiome

## Abstract

The effect of *Bartonella henselae* on the microbiome of its vector, *Ctenocephalides felis* (the cat flea) is largely unknown, as the majority of *C. felis* microbiome studies have utilized wild-caught pooled fleas. We surveyed the microbiome of laboratory-origin *C. felis* fed on *B. henselae*-infected cats for 24 h or 9 days to identify changes to microbiome diversity and microbe prevalence compared to unfed fleas, and fleas fed on uninfected cats. Utilizing Next Generation Sequencing (NGS) on the Illumina platform, we documented an increase in microbial diversity in *C. felis* fed on *Bartonella*-infected cats for 24 h. These changes returned to baseline (unfed fleas or fleas fed on uninfected cats) after 9 days on the host. Increased diversity in the *C. felis* microbiome when fed on *B. henselae*-infected cats may be related to the mammalian, flea, or endosymbiont response. Poor *B. henselae* acquisition was documented with only one of four infected flea pools having *B. henselae* detected by NGS. We hypothesize this is due to the use of adult fleas, flea genetic variation, or lack of co-feeding with *B. henselae*-infected fleas. Future studies are necessary to fully characterize the effect of endosymbionts and *C. felis* diversity on *B. henselae* acquisition.

## 1. Introduction

Despite its ability to transmit multiple zoonotic pathogens to humans and animals, *Ctenocephalides felis* (the cat flea) is poorly studied as a vector [1]. The most pathogenic *C. felis* transmitted bacterium is *Bartonella henselae*, the primary causative agent of cat scratch disease [2]. Infection with *B. henselae* is difficult to diagnose using culture, serology, or molecular methods due to the organism’s immune evasive mechanisms as well as the low levels of bacteremia in humans, dogs, horses, and other diseased non-reservoir hosts [3]. In a subset of bacteremic individuals, therapeutic elimination of *B. henselae* is exceptionally difficult to achieve due to pathogen evasive strategies (diverse virulence factors, intracellular niche, biofilm formation, widespread tissue distribution within the infected host, and immunomodulatory mechanisms), incomplete antibiotic efficacy, and the lack of available targeted therapeutics [4,5,6,7]. Therefore, preventing *B. henselae* transmission and subsequent infection is critical to both animal and human health. Disrupting the transmission of vector-borne diseases requires an understanding of the complex systems in which the vector and pathogen(s) are maintained and transmitted [8]. The vector microbiome represents a growing area of research that aims to assess the effects and interactions of pathogenic and nonpathogenic microorganisms on the survival, proliferation, and transmission of vector-borne pathogens [9,10]. These research efforts have given rise to control efforts for arboviruses and a greater understanding of the expanding epidemiology of several tick-transmitted pathogens [11,12].

Unfortunately, the *C. felis* microbiome remains incompletely described with reports mostly limited to wild-caught fleas, often with unknown hosts and feeding histories [13,14]. Wild-caught *C. felis* are primarily colonized by the genera *Bartonella, Rickettsia*, and *Wolbachia. Rickettsia* spp. associated with *C. felis* are primarily those in the transitional or *Rickettsia felis*-like organism group (RFLO), including *Rickettsia felis, Rickettsia asembonensis*, and ‘*Candidatus* Rickettsia senegalensis’ [13,14,15,16]. Three strains of *Wolbachia* have currently been associated with *C. felis*: wCfeT, wCfeJ, and wCfeF [17,18]. *Wolbachia* spp. display strain-specific effects on their host with many variants acting as extraordinary manipulators of insect biology including effects on reproduction (e.g., parthenogenesis and cytoplasmic incompatibility) and bacterial acquisition via cholesterol competition and insect immune modulation [19,20,21]. The effect of *Rickettsia* and *Wolbachia* infection in *B. henselae* acquisition, proliferation, and transmission by *C. felis* is largely unknown.

Previous studies tracking the dynamics of *B. henselae* establishment and proliferation within *C. felis* document decreasing bacterial loads over the first two days of flea feeding [22,23]. During this period (24 h), the *C. felis* immune system is believed to contribute to a period of bacterial purging. Subsequently, *B. henselae* bacterial loads are known to consistently increase up to the 9 day time point [22].

In order to investigate the effect of *B. henselae* on the *C. felis* microbiome, we utilized Next Generation Sequencing (NGS) of the 16S rRNA gene to assess the *C. felis* microbiome response to blood feeding and host *B. henselae* infection status. Our first objective was to compare the microbial diversity of *C. felis* before and after blood feeding for 24 h or 9 days. As a second objective, we assessed changes to the *C. felis* microbiome caused by feeding on *B. henselae*-infected versus uninfected cats. To accomplish these objectives, we performed 16S NGS of pooled *C. felis* prior to blood feeding or after 24 hrs or 9 days of blood feeding on naïve (uninfected) or *B. henselae*-infected cat. 

## 2. Materials and Methods

### 2.1. Cat Bartonella henselae Infection and C. felis Feeding

*Ctenocephalides felis*, cats, and *Bartonella henselae* (CSU Bh-1 Strain) acquisition and cat infection was performed as described in André et al. [24]. The study was reviewed and approved by Kansas State University KSU IACUC #4511-VMS and High Quality Research (HQR), Fort Collins, CO, USA (number #170.059). All four young female cats were housed in an ectoparasite flea facility at High Quality Research (Fort Collins, CO, USA) and fed a dry maintenance cat food. Cats underwent physical exam and qPCR to confirm overall health and *Bartonella* spp. naïve infection status, respectively. Four weeks prior to *C. felis* infestation, two of the cats were intradermally inoculated with sterile saline (cats #3363 and #3508) and two of the cats were inoculated with approximately 1.5 × 10^8^ *B. henselae* (cats #3320 and #3711). Experimental infection reduced the risk of vector-borne coinfection and allowed flea infection at the peak of bacteremia. Blood and serum collected from each cat on a weekly basis was utilized for qPCR and immunofluorescence antibody testing (IFA) to assess pathogen presence and *B. henselae* seroreactivity, respectively. Bacteremia was confirmed with culture isolation using blood agar plates. 

**Figure 1:**
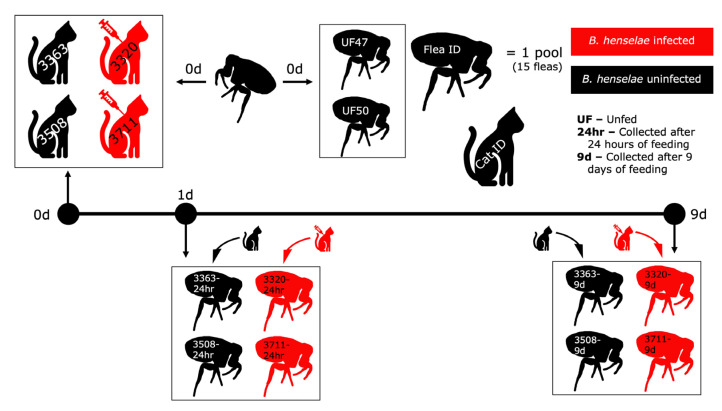
Study timeline beginning with the introduction of *C. felis* to the cats (day 0). Flea pools (15 fleas per cat per time point) were collected and are represented by a single flea with the sample number indicated. Red indicates a *B. henselae* infected cat or fleas from an infected cat. Bartonella henselae infection of cats occurred 4 weeks prior to introducing fleas onto the host cats.

*Ctenocephalides felis* were obtained from the Kansas State University (KSU, Manhattan, KS, USA) laboratory colony and raised as described previously [24]. The *C. felis* used to supply and replenish the KSU flea colony were obtained from the greater Manhattan, KS area. In short, *C. felis* eggs were obtained from adults feeding on seven cats. Eggs were incubated in a Petri dish at 28 °C and 70% relative humidity. Larvae and pupae were sifted from the media and placed into jars to emerge into adults; after that they were collected and transported to the animal facility for our study. At time 0, *C. felis* were placed on the cats. Each cat had 340 *C. felis* divided in two chambers attached to their shaved thorax and flank. For microbiome analysis, two groups of 15 *C. felis* were collected prior to being placed on a cat and served as the unfed, uninfected controls. At 24 h and 9 days after placement onto each cat, 15 *C. felis* per cat were collected and pooled [24]. The study timeline is depicted in Figure 1.

Following collection, each *C. felis* pool underwent four washes, two with PBS and two with ethanol. The fleas were then crushed using liquid nitrogen and a new sterile pestle for each group (Fisherbrand Pellet Pestle Cordless Motor). To avoid DNA carryover, DNA extraction and molecular testing were performed at separate benches. DNA was extracted using the Qiagen DNeasy Blood and Tissue Kit (Qiagen, Valencia, CA, USA) following the manufacturer’s tissue extraction protocol. Three 200 μL PBS controls (crushing controls) were introduced prior to crushing using liquid nitrogen and three 200 μL PBS controls (reaction controls) were introduced prior to the DNA extraction phase. These controls will henceforth be referred to as negative controls and served to identify contaminants from reagents that may be amplified during NGS [25].

### 2.2. PCR Assays

Detection of *B. henselae* was assessed via qPCR targeting the 16S–23S intergenic spacer (ITS) region [5]. Flea phylogeny was assigned utilizing PCR of the *cox*1 gene with the Cff-F and Cff-R primers according to previously published conditions [26]. Following amplification and sequencing, flea *cox*1 sequences were compared to the haplotypes and clades defined by Lawrence et al. [27]. 

### 2.3. Library Preparation and Sequencing 

DNA samples and controls were submitted to the North Carolina State University Genomic Sciences Laboratory for 16S rRNA amplification, bead cleanup with AMPure XP beads, index PCR, and Next Generation Sequencing targeting the V3–V4 region on the Illumina MiSeq platform. This region was selected using primers designed by Klindworth et al.: forward primer 5′-TCGTCGGCAGCGTCAGATGTGTATAAGAGACAGCCTACGGGNGGCWGCAG-3′ and the reverse primer 5′-GTCTCGTGGGCTCGGAGATGTGTATAAGAGACAGGACTACHVGGGTATCTAATCC-3′ and generated an approximately 402-base-pair sequence [28].

### 2.4. Data Analysis

Data analysis was performed in R. We first utilized the DADA2 pipeline (version 1.20.0) to visualize quality profiles, filter, and trim sequences then infer amplicon sequence variants (ASVs) and remove chimeras. The DADA2 assignTaxonomy function and non-redundant Silva taxonomic training database version 138.1 (“silva_nr99_v138.1_train_set.fa”, https://www.arb-silva.de/ (accessed on 3 May 2021)) were then utilized to assign sequence taxonomy to the genus level. 

We first attempted to utilize the decontam frequency and prevalence methods under default conditions. The decontam frequency method compares the abundance of ASVs in samples (including negative controls) with respect to sample DNA concentration following NGS sequencing library preparation. The decontam prevalence method compares the presence of ASVs in true samples to the presence in negative controls, regardless of ASV abundance. Given our low biomass, the decontam frequency method was not able to appreciate the difference between our negative control and true samples while the prevalence method did not account for differential ASV abundance. Therefore, we implemented an alternative filtering method in two steps: the first phase of filtering utilized negative control samples and is summed up by the equation below, where *a* is the abundance of reads in either true samples or negative controls and *n* is the number of true samples or negative controls containing that ASV. When γ is greater than 1, samples were considered true sequences, and when γ is less than 1, samples were removed as contamination. Secondly, ASVs accounting for fewer than 0.001% of reads in true samples were first removed.

$$\gamma =\frac{\sum a_{true\;samples}/n_{true\;samples}}{\sum a_{negative\;controls}/n_{negative\;controls}}$$

Following the removal of contaminants, diversity indices were calculated via the vegan package [29]. Sample richness, the number of different taxa, and Pielou’s evenness were calculated. Pielou’s evenness is represented by a number between 0 and 1 with a lower number indicating a more uneven distribution of species and a higher number indicating a more even community. Sample alpha diversity was calculated via the Shannon index and inverse Simpson index [30]. The Simpson index places greater weight on the evenness of species distribution while the Shannon index equally weighs richness and evenness. Statistical significance of sample diversity was compared using a standard t-test based on *B. henselae* status and time spent on cat.

Identification of specific ASVs associated with the flea fed status, cat *Bartonella* infection status, or time spent on the cat host was accomplished via the DEseq2 package in R utilizing the LRT test with an alpha of 0.01 [31].

## 3. Results

### 3.1. NGS Filtering

**Figure 2:**
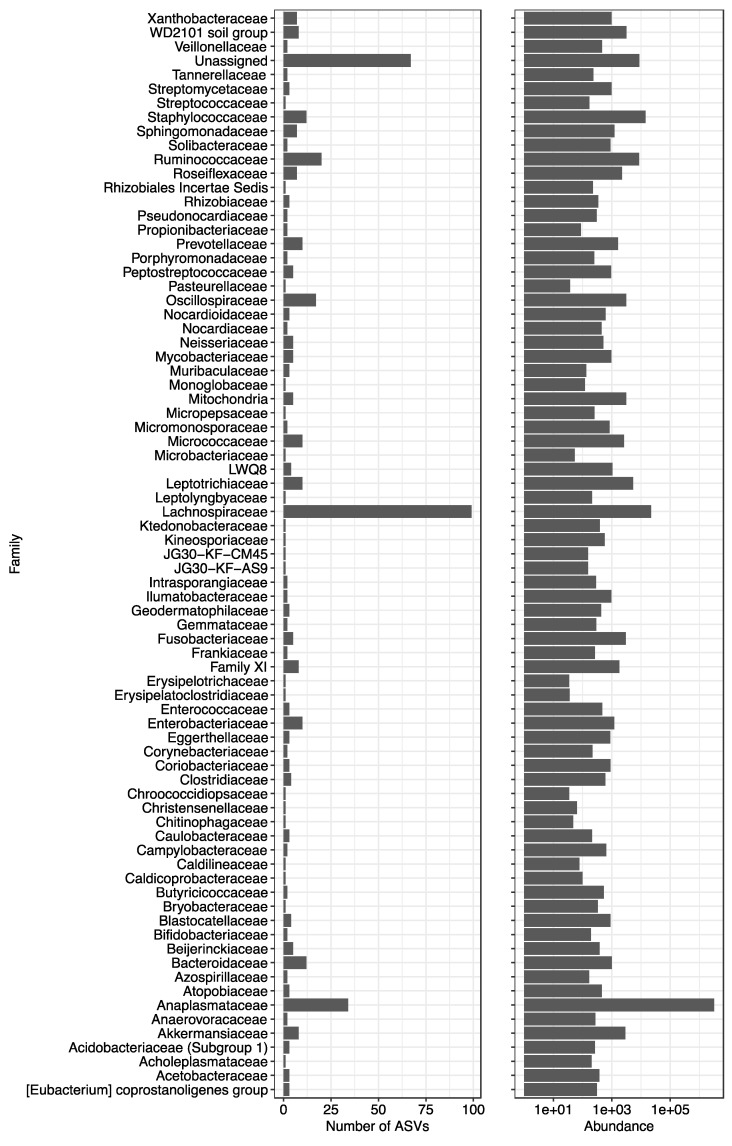
Bar chart displaying the number of Amplicon Sequence Variants (ASVs) assigned to each family and the abundance of reads (log-scale) assigned to a family from all flea samples. Reads which were unable to be assigned to the family level were designated as Unassigned.

NGS library preparation and application of the DADA2 pipeline identified 2419 ASVs from the ten flea pools and eight control samples with a minimum library size of 96,079 reads for control samples and 152,708 reads for flea samples. All filtering methods excluded the 1198 reads detected only in negative controls and retained the 882 ASVs detected only in true samples. Application of our novel filtering method identified 57% of ASVs (1386/2419) as contaminants due to their increased abundance in negative controls compared to flea pools. An additional 550 ASVs did not have an abundance over 0.001% of total reads, leaving 483 non-contaminant ASVs belonging to 58 orders, 77 families, and 114 genera (Figure 2). All detected genera and the proportion of reads assigned to each genus are available in Supplementary Table S1. The decontam prevalence and frequency method identified 1276 (53%) and 1206 (50%) ASVs as contaminants, respectively (Supplementary Figure S1). All contaminants identified by the prevalence method were also identified by our novel method. The decontam frequency method identified four contaminant ASVs not considered contaminants by our filtering or the prevalence method. As the frequency method did not separate the negative control and true samples, we opted to retain the four ASVs that were filtered out by the frequency method but not our method as these sequences were considered true sequences by both our filtering and the decontam prevalence method: ASV630 family Enterobacteriaceae, ASV885 family Bifidobacteriaceae, ASV925 genus *Prevotella*, and ASV1617 genus *Bacteroides*. 

### 3.2. Length of Feeding

**Figure 3:**
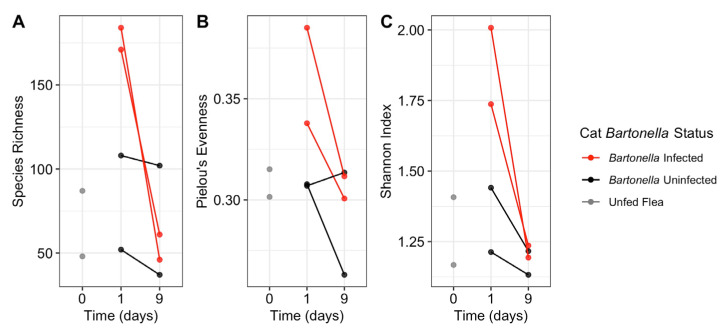
The total number of species (species richness, (**A**)), the evenness of samples with a lower number indicating a more uneven community (Pielou’s evenness, (**B**)), and alpha diversity (Shannon index, (**C**)) separated by time of sampling (x axis) and cat B. henselae infection status (color). Samples from the same cat are connected by a line.

**Figure 4:**
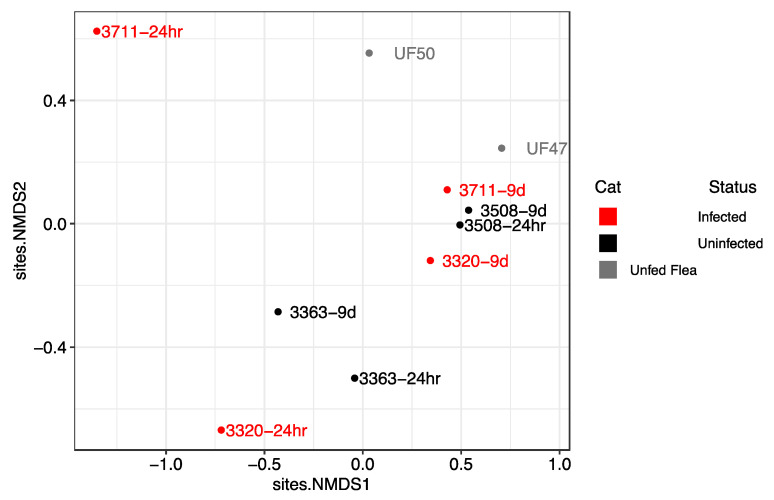
Non-metric Multidimensional Scaling (NMDS) plot of Next Generation Sequencing data to visualize sample similarity on a two dimensional plane. Samples are labelled with the cat number (#3363; #3508; #3320; #3711) and time point (24 h vs. 9 days) or unfed (UF), with color (red vs. black) indicating cat host *Bartonella* infection status (infected vs. uninfected).

Flea pools from all cats displayed a decrease in alpha diversity from 24 h to 9 days; however, this finding was not statistically significant (*p* = 0.10, Figure 3C). This decrease in alpha diversity is due to a decrease in both species richness and evenness for a majority of pools (Figure 3A,B). An NMDS plot did not consistently separate samples by fed status or time spent on a cat (Figure 4). 

**Figure 5:**
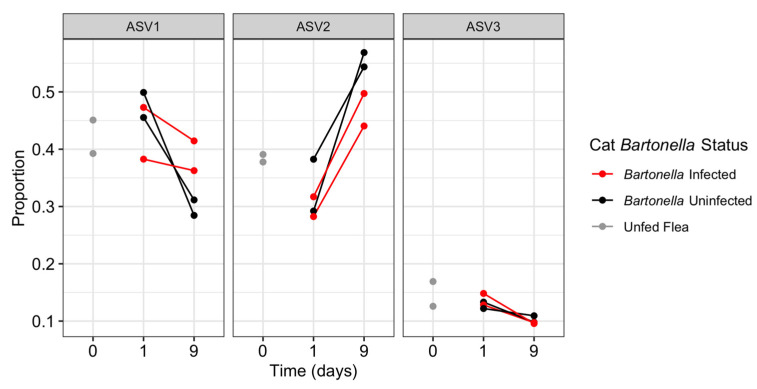
Proportion of reads per sample assigned to the three dominant *Wolbachia* ASVs by time point (unfed; 24 h; 9 days) and cat *B. henselae* status (infected vs. uninfected). Samples from the same cat are connected by a line.

The three most prevalent ASVs (all *Wolbachia* ASVs) displayed an inconsistent abundance response relative to the time fleas remained on a cat. In flea pools from all cats regardless of *B. henselae* status, ASV1 and ASV3 decreased in proportion of reads from 24 h to 9 days, whereas ASV2 increased in proportion of reads (Figure 5). 

### 3.3. Host Bartonella Status

On the basis of the Shannon and Simpson alpha diversity index, flea pools fed on *Bartonella*-infected cats for 24 h displayed greater diversity than fleas fed on uninfected cats (*p* = 0.09), which decreased by the 9 day time point (Figure 3C). This was accompanied by an increase in both species richness, and community evenness (Figure 3A,B). NMDS indicated that the flea pools fed on *B. henselae*-infected cat hosts for 24 h were distinct from other flea pools but were not similar to one another (Figure 4). By the 9 day time point, fleas fed on the *B. henselae*-infected cats clustered with flea pools from one uninfected cat (3508). Host *B. henselae* status did not influence the proportion of the three most abundant ASVs (all *Wolbachia* ASVs) over time (Figure 5). 

### 3.4. Bacterial Detection

One *Bartonella* spp. ASV (ASV696) was identified from a single flea pool (cat 3711 at time point 24 h) with relatively low abundance (122 reads, 0.05%). qPCR *amplified B. henselae* DNA from the 24-h and 9 day flea pools from cat 3711 and 3320 (Table 1). Fleas from cat 3711 had consistently higher *B. henselae* concentration as indicated by lower cycle threshold values.

*Rickettsia* ASVs were not detected in any flea pool, at any time point.

Of the 483 ASVs derived from all flea pools, 34 (7%) ASVs and 96.66% of reads were assigned to the family Anaplasmataceae and genus *Wolbachia*, the dominant genus in all flea pools. Eleven *Wolbachia* ASVs were detected in all flea pools including the three most prevalent *Wolbachia*: ASV1, ASV2, and ASV3. ASV1 displayed 100% homology with *Wolbachia* strain wCfeT (GenBank accession number CP051156.1, 402/402), ASV2 displayed 100% homology with *Wolbachia* strain wCfeF (GenBank accession number CP116767.1, 402/402), and ASV3 displayed 100% homology with *Wolbachia* strain wCfeJ (GenBank accession number CP051157.1, 402/402). 

### 3.5. Flea Cox1 Haplotypes

The portion of the *cox*1 gene we amplified was identical across flea pools (GenBank accession number MG668605.1, 479/479). When compared to the Lawrence et al. dataset, our *C. felis* resided within Clade 6, a tropical clade [27].

### 3.6. Differential Sequence Abundance Analysis

Differential sequence abundance analysis of all ASVs that were detected in two or more flea pools did not identify any specific ASV that was associated with fed status, time spent on the cat, or cat *B. henselae* infection status.

## 4. Discussion

In this study, there was an early increase in the *C. felis* microbiome richness, evenness, and alpha diversity when fleas were fed on *B. henselae*-infected cats for 24 h, followed by a return to that of unfed *C. felis* or *C. felis* fed on uninfected cats by the 9 day time point. Similarly, a Non-Metric Multidimensional Scaling (NMDS) plot of the study samples indicated that pooled *C. felis* fed on *Bartonella*-infected cats for 24 h were distinct from other samples at the 24 h time point, but clustered with samples from a *B. henselae* naïve cat (3508) at the 9 day time point. We hypothesize that the increase in *C. felis* microbiome diversity in response to feeding on a *B. henselae*-infected host may be due to factors that transiently suppressed the cat or *C. felis* immune system [24,32,33]. The decrease in microbial diversity in all fleas from the 24 h to 9 day time point (regardless of *B. henselae* infection) may be attributed to a lag in colonization resistance conveyed by resident microbes (e.g., *Wolbachia*) or the response of the *C. felis* immune system, which is known to be activated by blood meal ingestion [34]. Additional investigation of the larger diversity of microbes detected in *C. felis* is warranted.

Next Generation Sequencing results identified a large diversity of *Wolbachia* spp. within the microbiome of laboratory fleas, including 34 distinct ASVs. It is important to note that *Wolbachia* coinfection with multiple known strains in individual *C. felis* is a phenomenon believed to primarily occur in laboratory fleas [17]. Due to pooling fleas, our study did not address the individual *C. felis* microbiome; however, the presence of three *Wolbachia* strains in approximately equal proportions within this population suggests that they may stably coexist. Blood feeding was shown to affect the prevalence of the three dominant *Wolbachia* ASVs regardless of the cat host *B. henselae* infection status, with ASV1 and ASV3 displaying a decrease in abundance over time and ASV2 displaying an increase in abundance over time. Given the results of this study, further investigations of *Wolbachia* dynamics following blood feeding, as well as the effect of *Wolbachia* strains on flea-borne pathogen colonization and proliferation within *C. felis*, are warranted, as is research utilizing *Wolbachia* for pathogen control, as in other vector species [12].

Next Generation Sequencing only identified *Bartonella* infection in one of four pools derived from fleas feeding on *Bartonella* infected cats, whereas *B. henselae* DNA was successfully amplified from all four pools by qPCR at low cycle threshold values (Table 1). This low detection by NGS and low cycle threshold by qPCR is surprising given the number of fleas within each pool when feeding on a host infected with *B. henselae.* Previous studies have primarily used artificial membrane feeding systems for the infection of *C. felis* with *B. henselae*: Bouhsira et al. tested pools of 20 fleas fed on an artificial membrane and were able to detect *B. henselae* in the majority of samples over a 13-day study period by qPCR [23]. In another artificial membrane study utilizing qPCR, Robinson et al. detected *B. henselae* in 86% of individual membrane fed *C. felis* [35]. A few studies have evaluated the prevalence of *B. henselae* in *C. felis* fed on known infected cats: Finkelstein et al., reported PCR detection in 66% (2/3) of flea pools with 20 fleas per pool when fed on cats naturally infected with *B. henselae* [36]. Despite inconsistent detection in laboratory fleas, wild-caught *C. felis* with unknown feeding history are regularly reported to be infected with *B. henselae* by NGS, whether pooled or submitted as individual fleas [13,14,26]. While *B. henselae* acquisition efficiency is known to be imperfect, given the flea pool sizes and confirmed cat bacteremia (via blood culture and qPCR) prior to and after infestation [24], we expected all flea pools to harbor *B. henselae* at levels detectable by NGS. 

We offer five preliminary hypotheses regarding the low *B. henselae* acquisition by *C. felis* in this study. (1) Vertical nontransovarial transmission of *Bartonella* spp. via the consumption of debris and flea feces by larval fleas, as documented in *Xenopsylla ramesis* fleas, may be more important for maintenance within the flea population than horizontal transmission from a cat to flea [37]. (2) Long term *C. felis* infestation may be necessary to trigger *Bartonella* host bacteremia in the quantities necessary for transmission or to facilitate localization in the cat’s skin to infect naïve *C. felis.* (3) Co-feeding with *Bartonella*-infected *C. felis* is necessary to achieve significant acquisition by naïve fleas. Horizontal transmission of *R. felis* between fleas co-feeding on an artificial system has been documented; however, the importance of co-feeding in the infection of the flea on host has not been established for *R. felis* or other flea-borne pathogens [38]. (4) *B. henselae* may display variable acquisition by *C. felis* dependent upon *C. felis* genetic diversity and the presence or absence of coinfecting bacteria (e.g., *Rickettsia felis*-like organisms). The fleas within this study were assigned to Clade 6, a clade typically found in the more humid and warm areas associated with *B. henselae*, such as the Southeastern United States [27,39]. (5) A large amount of flea DNA may have overwhelmed *B. henselae* DNA resulting in poor amplification. Unfortunately, due to the low biomass of our samples, dilution for NGS was considered disadvantageous. 

The insect immune system functions via various cellular and humoral mechanisms. Within the digestive tract, serine proteases function in blood meal digestion and have been implicated in the cat flea immune response to *B. henselae* [24] and *R. typhi* [40]. Phosphoenolpyruvate carboxykinase, succinic semialdehyde dehydrogenase (SSADH), and secreted salivary acid phosphatase are also associated with *B. henselae* ingestion by cat fleas, with a known role in responding to environmental stress, energy production, and preventing homeostasis, respectively [24]. Further exploration of their role in the immune response is warranted. Phagolysozyme mediated destruction is documented following *C. felis* ingestion of *R. felis;* however, it is unknown if this occurs with *B. henselae* [41]. Among the most common insect immune pathways, all Imd and Toll pathway genes have been identified within the *C. felis* genome with the Imd pathway known to modulate *R. typhi* infection [42]. Further research regarding the *C. felis* immune system’s effect on pathogenic and non-pathogenic bacteria is necessary.

As mentioned above, various microbiome members beyond *Bartonella* and *Wolbachia* spp. were detected. The four most abundant families were the Anaplasmataceae, Lachnospiraceae, Staphylococcaceae, and Ruminococcaceae. The Anaplasmataceae family was exclusively represented by *Wolbachia* spp. The Lachnospiraceae family, with the highest number of distinct ASVs (*n* = 99), as well as the Ruminococcaceae family, are associated with the digestive process in mammals and insects alike suggesting a similar role in *C. felis* [43,44]. Staphylococcaceae are regularly reported in other blood-sucking insects [45] and are believed to be acquired via habitat or host skin contamination [46]. 

Our alternative NGS ASV filtering method identified 1386 ASVs as contaminants on the basis of higher abundance in negative controls than infected true samples. Both methods implemented by decontam identified fewer ASVs as contaminants under default conditions. Increasing the stringency of decontam filtering would increase removal of contaminants yet not address the abundance differences in true samples and negative controls. Given the low biomass of our samples, the decontam frequency method was not able to separate true samples and negative controls based on DNA concentration. The decontam prevalence method does not consider the abundance of ASVs in true samples versus negative controls and is best employed for sample types with a dominance of non-contaminant ASVs. Therefore, we suggest that our filtering method is better suited to the present sample set that includes low biomass samples and low sample number. 

Differential sequence abundance analysis did not identify any ASVs as significantly associated with fed status, time fleas spent on a cat, or the cat *B. henselae* infection status. This may be attributed to the small number of replicates or utilization of laboratory-raised *C. felis* that lack microbiome members observed in wild-caught fleas that may be important for the acquisition of pathogens. 

The limitations of the current study include the small sample size (number of cats and flea pools), which limited the power of statistical techniques utilized for analysis. Furthermore, as our *C. felis* were pooled together, we were unable to draw conclusions regarding the coinfection of specific bacteria within the individual *C. felis* microbiome. However, this pooling was necessary to acquire sufficient biomass for NGS. Additionally, the laboratory setting fails to recapitulate the complexity of *C. felis* exposure to diverse host species, genetic diversity among *C. felis* strains, and full diversity of microbiome members. Finally, the artificial cat infection model used may not accurately mimic the events occurring in natural *C. felis–B. henselae* infection, including microbiome changes. Future laboratory and field work is necessary to further elucidate the effect(s) of specific microbiome members in the response of *C. felis* to feeding on a *B. henselae*-infected host. 

## 5. Conclusions

In conclusion, this study documented an increased *C. felis* microbial diversity when fed for a limited time (24 h) on cats infected with *B. henselae*, with diversity returning to that of unfed *C. felis* or *C. felis* fed on uninfected cats by the 9 day time point. This finding may indicate altered response by the *C. felis* immune system or microbiome when ingesting *B. henselae*. Despite the increase in microbial diversity, *B. henselae* infected *C. felis* at a low abundance compared to wild-caught or previous laboratory *C. felis* studies. We confirmed *Wolbachia* spp. as the dominant member within the laboratory *C. felis* microbiome with three strains dominating all *C. felis* pools. These *Wolbachia* strains appeared to respond differently to long-term blood-feeding, with two strains decreasing and one strain increasing in abundance over time. To assess if *C. felis* feeding influences pathogen acquisition by naïve *C. felis*, future research should examine the effect of long-term *C. felis* colonization of the cat host in relation to *B. henselae* bacteremia and bacterial skin localization in the cat. *Ctenocephalides felis* genetic variation is an exciting new frontier that may facilitate a deeper understanding of pathogen spread and the extent to which vector diversity influences epidemiology and pathogen transmission. Finally, examining the effect of pathogenic and non-pathogenic bacterial coinfection of *C. felis* via laboratory and field studies presents an opportunity to better understand and potentially interrupt *B. henselae* transmission by the flea vector, thereby preventing bartonellosis in animals and humans.

## Figures and Tables

**Table 1:** *Bartonella* spp. qPCR results of flea samples by cat and time point (24 h or 9 days). Indicated as + (positive) or – (negative) with qPCR cycle threshold indicated in parentheses.

Sample	Host Cat *B. henselae* Status	ITS qPCR (Ct)
3711-24 h	Infected	+ (31.61)
3711-9 d	Infected	+ (32.48)
3320-24 h	Infected	+ (38.13)
3320-9 d	Infected	+ (41.48)
3363-24 h	Naïve	− (N/A)
3363-9 d	Naïve	− (N/A)
3508-24 h	Naïve	− (N/A)
3508-9 d	Naïve	− (N/A)

## Data Availability

The datasets utilized in this study are available at https://doi.org/10.5061/dryad.ncjsxkszn.

## Supplementary Materials

The following supporting information can be downloaded at: https://www.mdpi.com/article/10.3390/pathogens12030366/s1. Figure S1: *p*-value assigned to ASVs by the Decontam prevalence method (A) or Decontam frequency method (B). If greater abundance was identified in infected positive controls or negative controls, the basis of our filtering (C). Table S1: all 114 genera reported by our filtering method as true sequences. The family, and proportion of reads from all flea pools assigned to these genera, is reported.
