# Supplementary material for: Feeding on a Bartonella henselae Infected Host Triggers Temporary Changes in the Ctenocephalides felis Microbiome
Source: Pathogens. 2023 Feb 22;12(3):366. doi: 10.3390/pathogens12030366 (PMC10056022; doi:10.3390/pathogens12030366)
Supplement: Supplementary file 1 [file pathogens-12-00366-s001.zip › pathogens-2147563-supplementary/SupplementaryTable1.pdf]

| Family | Genus | Proportion |
|---|---|---|
| **Anaplasmataceae** | Wolbachia | 98.0412 |
| **Fusobacteriaceae** | Fusobacterium | 0.0942 |
| **Ruminococcaceae** | Incertae Sedis | 0.1748 |
| **Family XI** | Peptoniphilus | 0.0305 |
| **Ruminococcaceae** | Faecalibacterium | 0.052 |
| **Leptotrichiaceae** | Leptotrichia | 0.156 |
| **Lachnospiraceae** | Roseburia | 0.0563 |
| **Akkermansiaceae** | Akkermansia | 0.0893 |
| **Lachnospiraceae** | Lachnoclostridium | 0.0733 |
| **Lachnospiraceae** | Oribacterium | 0.0461 |
| **Ruminococcaceae** | UBA1819 | 0.0217 |
| **Micrococcaceae** | Rothia | 0.0677 |
| **Kineosporiaceae** | Quadrisphaera | 0.0176 |
| **Lachnospiraceae** | [Ruminococcus] torques group | 0.02 |
| **Mycobacteriaceae** | Mycobacterium | 0.0298 |
| **Solibacteraceae** | Candidatus Solibacter | 0.028 |
| **Coriobacteriaceae** | Collinsella | 0.0278 |
| **Prevotellaceae** | Prevotella_7 | 0.0301 |
| **Lachnospiraceae** | Lachnospiraceae NK4A136 group | 0.0634 |
| **Micromonosporaceae** | Actinoplanes | 0.0135 |
| **Peptostreptococcaceae** | Peptostreptococcus | 0.0242 |
| **Blastocatellaceae** | Tellurimicrobium | 0.013 |
| **Lachnospiraceae** | Blautia | 0.0551 |
| **Oscillospiraceae** | Oscillibacter | 0.0409 |
| **Butyricicoccaceae** | Butyricicoccus | 0.0164 |
| **Veillonellaceae** | Veillonella | 0.0124 |
| **Lachnospiraceae** | GCA-900066575 | 0.0155 |
| **Lachnospiraceae** | [Eubacterium] fissicatena group | 0.0258 |
| **Ktedonobacteraceae** | 1959-1 | 0.012 |
| **Lachnospiraceae** | Lachnospiraceae UCG-004 | 0.0118 |
| **Campylobacteraceae** | Campylobacter | 0.0202 |
| **Lachnospiraceae** | Acetatifactor | 0.0114 |
| **Leptotrichiaceae** | Streptobacillus | 0.0109 |
| **Sphingomonadaceae** | Hephaestia | 0.0104 |
| **Bryobacteraceae** | Bryobacter | 0.0103 |
| **Oscillospiraceae** | UCG-005 | 0.0102 |
| **Family XI** | Anaerococcus | 0.0217 |

| | | |
|---|---|---|
| **Eggerthellaceae** | Eggerthella | 0.0186 |
| **Nocardiaceae** | Rhodococcus | 0.0139 |
| **Streptomycetaceae** | Streptomyces | 0.0166 |
| **Oscillospiraceae** | Colidextribacter | 0.0205 |
| **Atopobiaceae** | Atopobium | 0.0121 |
| **Xanthobacteraceae** | Bradyrhizobium | 0.0157 |
| **Eggerthellaceae** | Enterorhabdus | 0.0089 |
| **Nocardioidaceae** | Marmoricola | 0.0188 |
| **Enterobacteriaceae** | Aquamonas | 0.0246 |
| **Sphingomonadaceae** | Sphingomonas | 0.0173 |
| **Lachnospiraceae** | Marvinbryantia | 0.0119 |
| **Pseudonocardiaceae** | Kibdelosporangium | 0.0081 |
| **Oscillospiraceae** | Flavonifractor | 0.0079 |
| **Lachnospiraceae** | [Eubacterium] hallii group | 0.0152 |
| **Intrasporangiaceae** | Terrabacter | 0.0077 |
| **Lachnospiraceae** | A2 | 0.0075 |
| **Lachnospiraceae** | Lachnoanaerobaculum | 0.0103 |
| **Sphingomonadaceae** | Altererythrobacter | 0.0074 |
| **Micrococcaceae** | Glutamicibacter | 0.0074 |
| **Clostridiaceae** | Clostridium sensu stricto 1 | 0.0187 |
| **Blastocatellaceae** | JGI 0001001-H03 | 0.007 |
| **Rhizobiales Incertae Sedis** | Alsobacter | 0.007 |
| **Frankiaceae** | Jatrophihabitans | 0.0082 |
| **Gemmataceae** | Zavarzinella | 0.0091 |
| **Lachnospiraceae** | Fusicatenibacter | 0.012 |
| **Bacteroidaceae** | Bacteroides | 0.0308 |
| **Acholeplasmataceae** | Anaeroplasma | 0.0063 |
| **Lachnospiraceae** | Sellimonas | 0.0058 |
| **Xanthobacteraceae** | Pseudolabrys | 0.0057 |
| **Acidobacteriaceae (Subgroup 1)** | Terracidiphilus | 0.0071 |
| **Geodermatophilaceae** | Geodermatophilus | 0.0101 |
| **Corynebacteriaceae** | Corynebacterium | 0.0053 |
| **Streptococcaceae** | Streptococcus | 0.0052 |
| **Micrococcaceae** | Acaricomes | 0.0052 |
| **Peptostreptococcaceae** | Romboutsia | 0.0051 |
| **Lachnospiraceae** | Catonella | 0.0049 |
| **Rhizobiaceae** | Aureimonas | 0.0047 |
| **Anaerovoracaceae** | Family XIII AD3011 group | 0.0045 |
| **Lachnospiraceae** | Anaerostipes | 0.0133 |

| | | |
|---|---|---|
| **Oscillospiraceae** | Intestinimonas | 0.0148 |
| **Porphyromonadaceae** | Porphyromonas | 0.0078 |
| **Bifidobacteriaceae** | Bifidobacterium | 0.0042 |
| **Anaerovoracaceae** | [Eubacterium] nodatum group | 0.0039 |
| **Caulobacteraceae** | Phenylobacterium | 0.0065 |
| **Rhizobiaceae** | Bartonella | 0.0038 |
| **Monoglobaceae** | Monoglobus | 0.0037 |
| **Acetobacteraceae** | Acidiphilium | 0.0036 |
| **Beijerinckiaceae** | Microvirga | 0.0091 |
| **Geodermatophilaceae** | Blastococcus | 0.0033 |
| **Caldicoprobacteraceae** | Caldicoprobacter | 0.0031 |
| **Lachnospiraceae** | [Ruminococcus] gnavus group | 0.0059 |
| **Sphingomonadaceae** | Porphyrobacter | 0.003 |
| **Lachnospiraceae** | Lachnospiraceae UCG-006 | 0.0029 |
| **Lachnospiraceae** | Dorea | 0.0027 |
| **Family XI** | Parvimonas | 0.0027 |
| **Azospirillaceae** | Skermanella | 0.0026 |
| **Azospirillaceae** | Azospirillum | 0.0026 |
| **Lachnospiraceae** | Lachnospiraceae UCG-001 | 0.0024 |
| **Rhizobiaceae** | Allorhizobium-Neorhizobium-Pararhizobium-Rhizobium | 0.0022 |
| **Atopobiaceae** | Olsenella | 0.0021 |
| **Christensenellaceae** | Christensenellaceae R-7 group | 0.002 |
| **Microbacteriaceae** | Clavibacter | 0.0017 |
| **Propionibacteriaceae** | Cutibacterium | 0.0016 |
| **Corynebacteriaceae** | Turicella | 0.0015 |
| **Lachnospiraceae** | Agathobacter | 0.0026 |
| **Xanthobacteraceae** | Afipia | 0.0014 |
| **Prevotellaceae** | Prevotella | 0.0026 |
| **Beijerinckiaceae** | Methylobacterium-Methylorubrum | 0.0027 |
| **Pseudonocardiaceae** | Pseudonocardia | 0.0013 |
| **Family XI** | Ezakiella | 0.0012 |
| **Intrasporangiaceae** | Pedococcus-Phycicoccus | 0.0012 |
| **Propionibacteriaceae** | Friedmanniella | 0.0012 |
| **Ruminococcaceae** | Candidatus Soleaferrea | 0.0012 |
| **Pasteurellaceae** | Conservatibacter | 0.0011 |
| **Erysipelatoclostridiaceae** | Erysipelatoclostridium | 0.0011 |
| **Acidobacteriaceae (Subgroup 1)** | Terriglobus | 0.0011 |

**Supplementary Table 1** all 114 genera reported by our filtering method as true sequences. The family, and proportion of reads from all flea pools assigned to these genera is reported.
