# Supplementary figures and images for: Feeding on a Bartonella henselae Infected Host Triggers Temporary Changes in the Ctenocephalides felis Microbiome
Source: Pathogens. 2023 Feb 22;12(3):366. doi: 10.3390/pathogens12030366 (PMC10056022; doi:10.3390/pathogens12030366)

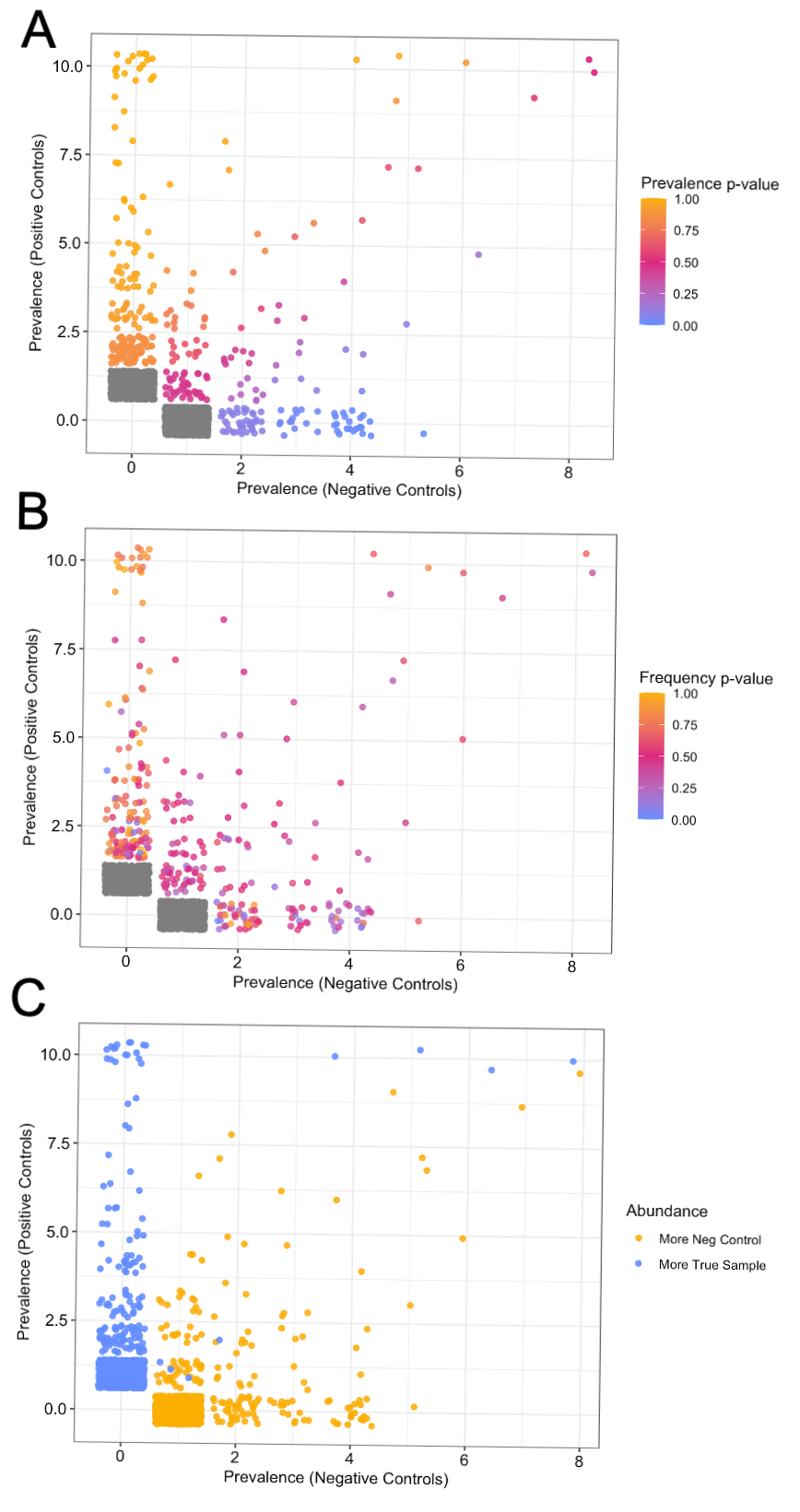

Supplement: Supplementary file 1 [file pathogens-12-00366-s001.zip › pathogens-2147563-supplementary/SupFig1.tif]
